# Effects of Reproductive Materials on Growth, Corm Yield, and Glucomannan Content in *Amorphophallus bulbifer*

**DOI:** 10.1002/fsn3.71131

**Published:** 2025-11-17

**Authors:** Xu Susu, Li Jinwei, Yuan Huifang, Ma Suyun, Liang Zhengwei, Yang Liping, Gong Yanxiong, Yan Suo, Xu Wenguo, Yan Xiangshuai

**Affiliations:** ^1^ Sichuan Yanbian KKonjac Biotech Co., Ltd Panzhihua China; ^2^ College of Agronomy and Biotechnology Yunnan Agricultural University Kunming China; ^3^ Yunnan Institute of Tropical Crops Xishuangbanna China; ^4^ Hong He University Honghe China; ^5^ Dehong Agricultural Science Research Institute Dehong China

**Keywords:** *araceae*, bulbils, morphological characteristics, net photosynthetic rate, tissue‐cultured seedlings

## Abstract

*Amorphophallus bulbifer*, known for its high glucomannan content, has extensive applications and significant economic value. However, the effects of different types of propagation materials on the yield and glucomannan content of *A. bulbifer* have not been thoroughly understood. This study compares the impacts of six propagation materials on the dynamic changes of morphological and physiological characteristics across three growth periods, as well as their effects on yield and the glucomannan content in corms in 2022 and 2023. The results indicated that the yield hierarchy among the six propagation materials was as follows: 2‐year‐old corms (T1) > 1‐year‐old corms propagated from bulbils (T3) > 1‐year‐old corms propagated from seeds (T5) > bulbils (T2) > tissue‐cultured seedlings (T6) > seeds (T4). The sizes of the aboveground morphological characteristics (petiole diameter, plant height, and leaf area) were consistently largest in the T1 treatment and smallest in the T4 treatment. Based on 2 years of data, in the new corm formation stage, chlorophyll content was highest in the T5 treatment, exceeding T4 by 49.36%. T1 had the highest soluble sugar content, surpassing T6 by 70.19%, while T2 had the highest soluble protein content, 110.45% higher than T5. In the bulking stage, T5 again had the highest chlorophyll content, 58.96% higher than T6. T6 showed the highest net photosynthetic rate, 24.74% higher than T2. T2 had the highest soluble sugar content, 114.91% more than T6, and T6 had the highest soluble protein content, surpassing T1 by 253.94%. At maturity, T4 had the highest chlorophyll and soluble protein levels, exceeding T3 by 74.80% and 134.71%, respectively. T6 had the highest net photosynthetic rate, 97.18% higher than T2. In summary, the 1‐year‐old corms propagated from seeds exhibited the highest glucomannan content while having a low weight, making it the optimal propagation material for *A. bulbifer* .

## Graphical abstract

This study compares six propagation materials for Amorphophallus bulbifer. One‐year‐old corms propagated from seeds are the optimal propagation material.

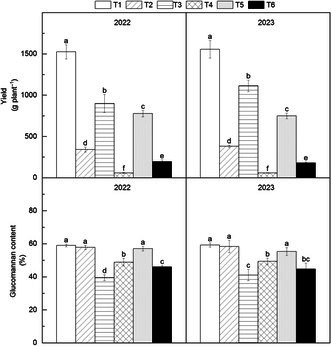

## 1 Introduction

*Amorphophallus*, a genus of perennial herbaceous plants belonging to the family Araceae, is primarily distributed in southern China, Southeast Asia, and South Asia (Li et al. 2024). The underground corm is the economic organ of *Amorphophallus*, rich in various chemical components, including glucomannan, proteins, amino acids, minerals, and alkaloids (Zhang et al. 2021). It is noteworthy that *Amorphophallus* is one of the few plants in nature that contains natural glucomannan (Behera and Ray 2017). Among cultivated plants, it stands out for its combination of wide planting range, strong environmental adaptability, and particularly efficient glucomannan extraction capabilities (Shi et al. 2019). As a food ingredient, glucomannan offers several benefits, including weight loss and satiety, oral health, enhancing the growth and viability of beneficial organisms in the colon, and binding of nutrients (e.g., cholesterol) (Tester and Al‐Ghazzewi 2017). In addition to its food uses, glucomannan shows therapeutic potential, acting as supplementary treatments for various conditions, including hemostasis, tumors, coughs, asthma, and diabetes (Kapoor et al. 2024). Modified glucomannan exhibits excellent water retention and gelation abilities, along with the capability to form films (Zhou et al. 2022). These functional characteristics make glucomannan an excellent ingredient for applications such as biodegradable films, medical and pharmaceutical materials, encapsulation and controlled release systems, fish feed, and cosmetics, among others (Zhu 2018). Due to the high moisture content (approximately 80%) of fresh *Amorphophallus* corms, they cannot be stored for extended periods after harvest (Tsehay et al. 2023). Consequently, the corms are typically processed into refined powder to facilitate storage and transportation (Kapoor et al. 2024). The refined *Amorphophallus* corms powder primarily consists of glucomannan, which accounts for 30% to 95% of its composition (Kapoor et al. 2024). Given the diverse applications of glucomannan and the established processing techniques for *Amorphophallus* corms, *Amorphophallus* holds considerable market potential and economic value.

According to the database maintained by the International Aroid Society, approximately 170 varieties of *Amorphophallus* are currently known worldwide, of which only a few have been domesticated and are considered edible (Behera and Ray 2017; Hetterscheid 2021). The main konjac species cultivated in China are *Amorphophallus konjac* and *Amorphophallus albus* (Chua et al. 2010). However, repeated cultivation of *A. konjac* and *A. albus* is prone to infections from soft rot, white rot, and root rot, which can result in reduced yields or even complete crop failure (Wu et al. 2012). *Amorphophallus bulbifer* is a species of *Amorphophallus* distinguished by its ability to produce bulbils on its leaves (Putu Laksmi Putri et al. 2023). The majority of *A. bulbifer* varieties are triploid, whereas *A. konjac* and *A. albus* are diploid (Zhao et al. 2020). The advantageous chromosome structure of *A. bulbifer* contributes to larger morphology and increased corm yield (Zheng et al. 2013). Notably, *A. bulbifer* exhibits stronger disease resistance, which offers promise for overcoming the bottlenecks associated with the susceptibility of *Amorphophallus* to soft rot, white root rot, and root rot diseases (Zheng et al. 2013). In addition, *A. bulbifer* corms are also rich in glucomannan, with even higher concentrations than those found in *A. konjac* and *A. albus* (Shenglin et al. 2020). Moreover, bulbils also exhibit reproductive abilities, enabling a more diverse array of reproductive strategies for *A. bulbifer* (Fialová et al. 2014).

The reproductive materials for *A. konjac* and *A. albus* include corms, stem cuttings, seeds, and tissue‐cultured seedlings (Li et al. 2023). Two‐year‐old corm, also known as large corm, can be utilized as reproductive material in two forms: either as whole bulbs or as sections (Qi et al. 2023). The use of 2‐year‐old corm as reproductive materials presents several disadvantages, such as the large quantity of corm required, susceptibility to pathogen infections, and high costs associated with storage, transportation, and planting (Shenglin et al. 2020). Seeds have a light weight, but when used as propagation material, the yield of *Amorphophallus* corms is low (Yangguang et al. 2023). One‐year‐old corms propagated from seeds, also known as small corm, have a moderate weight and are currently considered an ideal propagation material (Xu et al. 2024). The weight of 1‐year‐old corms propagated from bulbils is similar to that of 1‐year‐old corms propagated from seeds. The technology for in vitro cultivation of *Amorphophallus* seedlings addresses several issues associated with traditional propagation materials, such as long breeding cycles, variable seedling quality, and degeneration of varieties during cultivation (Li et al. 2021). Despite its advantages, research on optimal propagation materials for *A. bulbifer* remains limited, particularly in comparison to *A. konjac* and *A. albus* . This study aims to address this gap by evaluating different propagation materials to optimize yield and glucomannan content.

**FIGURE 1:**
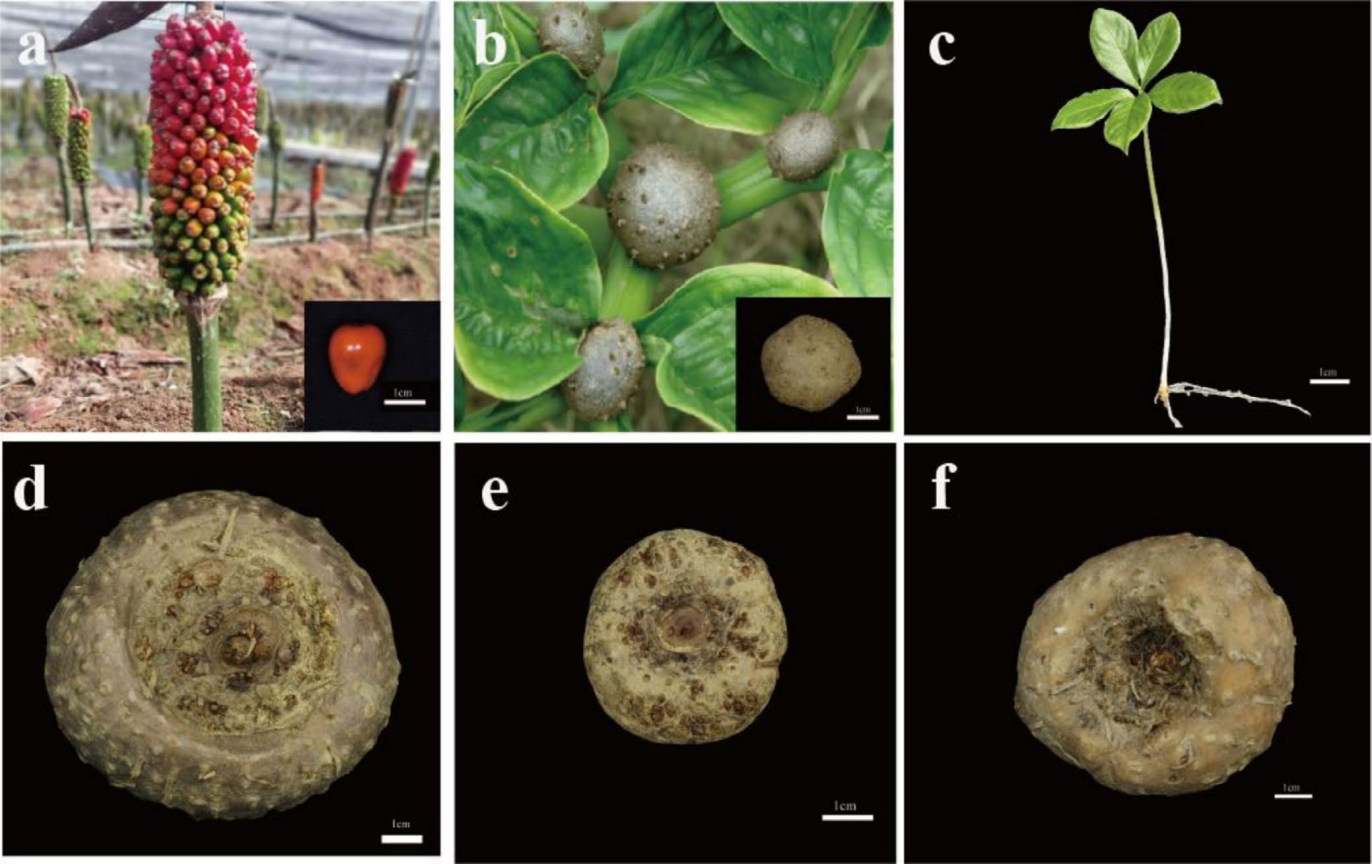
Reproductive materials of six types of *Amorphophallus muelleri*. (a) Seeds, (b) Bulbils, (c) Tissue cultured seedlings (TCS), (d) Two‐year‐old corms (TYC), (e) One‐year‐old corms propagated through seeds (TYC‐S), (f) One‐year‐old corms propagated through bulbils (TYC‐B).

Despite the economic importance of *A. bulbifer* , systematic studies evaluating the impact of diverse propagation materials on its agronomic traits and glucomannan accumulation are lacking. Understanding how material source affects physiological processes and ultimately glucomannan yield is critical for optimizing production. We hypothesize that: (1) Heavier propagation materials (e.g., 2‐year‐old corms) will provide greater initial nutrient reserves, leading to superior early growth and higher final corm yield; (2) The developmental stage and origin of the material (seed‐derived vs. bulbil‐derived corms) will significantly influence metabolic efficiency and glucomannan synthesis. To test these hypotheses, we selected six propagation materials representing key reproductive strategies: seeds (Figure 1a), bulbils (Figure 1b), tissue‐cultured seedlings (Figure 1c), 2‐year‐old corms (Figure 1d), 1‐year‐old corms propagated from seeds (Figure 1e), and 1‐year‐old corms propagated from bulbils (Figure 1f). This design allows direct comparison of material age, source, and propagation method.

## 2 Materials and Methods

### 2.1 Experimental Site

The experiment was conducted in 2022 and 2023 at the Yunnan Institute of Tropical Crop farm, Xishuangbanna County, Yunnan Province, China (22°00′49″ N, 100°45′51″ E). The experiment site is located at an altitude of 590 to 760 m and is classified as a north subtropical southwestern monsoon climate, with an average annual temperature of 21.5°C and an annual rainfall of 1161.8 mm. The soil at the experimental site (0–20 cm) exhibits the following characteristics: organic matter content of 29.13 g kg^−1^, alkaline hydrolysable nitrogen of 58.6 mg kg^−1^, available phosphorus of 18.41 mg kg^−1^, available potassium of 137.5 mg kg^−1^, and pH of 5.34.

### 2.2 Experimental Treatments and Design

The experiment was conducted using a single‐factor randomized block design, comprising six types of reproductive materials for *Amorphophallus bulbifer*: 2‐year‐old corms (T1), bulbils (T2), 1‐year‐old corms propagated from bulbils (T3), seeds (T4), 1‐year‐old corms propagated from seeds (T5), and tissue‐cultured seedlings (T6). Specifically: Source materials for 2022: T1, T2, and T4 were derived from 2‐year‐old corms, bulbils, and seeds (respectively) propagated from 1‐year‐old corms planted in 2021. T6 tissue‐cultured seedlings were cultivated from 1‐year‐old corms via in vitro methods. T3 and T5 corms were obtained from bulbils and seeds planted in 2021. Consistency in 2023: The same sources and propagation methods were used as in 2022. Each treatment was replicated three times, with plot dimensions of 3.9 × 10.0 m.

All six types of reproductive materials were sown using a hole planting method, with a plant spacing of 40 × 50 cm. Each hole contained one bulb, seed, or tissue‐cultured plant, with a sowing depth of 8 cm. All six types of reproductive materials were planted annually on May 12. Prior to sowing, chicken manure organic fertilizer (organic matter: 25.5%, N: 1.63%, P_2_O_5_: 1.54%, K_2_O: 0.85%) was applied at a rate of 15 t ha^−1^ as a base fertilizer. After fertilization, furrows were established to create ridges that measured 1.2 m wide and 30 cm high, with a furrow width of 30 cm. In the new corm formation period of *Amorphophallus*, a compound fertilizer (N:P_2_O_5_:K_2_O = 15:15:15) was applied at a rate of 300 kg ha^−1^. In the bulking period, a different compound fertilizer (N:P_2_O_5_:K_2_O = 15:5:25) was also applied at the same rate of 300 kg ha^−1^. Soil moisture levels were monitored using a soil moisture sensor (LD‐Y485, Lande, Jinan, China) to ensure timely irrigation, maintaining field moisture content between 40% and 50%. Additionally, prompt measures were taken to manage pests and diseases to prevent yield loss.

The growth period of konjac was divided into three stages: (1) New corm formation stage: After planting, nutrients from the reproductive material decompose rapidly, supporting root, aerial, and new corm development. This stage ends when the first leaf fully expands, marking the onset of the head‐changing period (Qi et al. 2023). (2) Bulking stage: Approximately 45 days after new corm formation, the corms begin to expand, lasting about 3 months. (3) Maturity stage: Defined as 10 days after the aerial parts collapse.

### 2.3 Measurement Items and Methods

#### 2.3.1 Morphological Characteristics

Each plot was marked with 10 *Amorphophallus* plants to determine morphological indicators. Measurements of petiole diameter, plant height, and leaf area were taken during the new corm formation phase, bulking phase, and maturation phase. The petiole diameter was measured 2 cm above the ground, while plant height was defined as the distance from the ground to the point where the petiole branches. The leaf area was defined as the sum of the areas of individual leaves, with the width of each leaf calculated as the average of its length and width.

#### 2.3.2 Physiological Characteristics

During the new corm formation phase, bulking phase, and maturation phase, five *Amorphophallus* plants were selected from each plot. The net photosynthetic rate (Pn) of fully expanded leaves from the middle canopy was measured using a gas exchange analyzer (Li‐6400, Li‐COR Inc., Lincoln, NE, USA) on a sunny day between 09:00 and 11:30 a.m., when the photosynthetic active radiation above the canopy reached 1200 mmol m^−2^ s^−1^ and the CO_2_ concentration was set at 400 μmol mol^−1^. Then, the collected leaves were mixed to create a composite leaf sample for that plot, swiftly preserved in liquid nitrogen, and subsequently transferred to the laboratory for storage at −80°C. Soluble sugar content was determined using the anthrone colorimetric method (Hamedalla et al. 2022). Soluble protein content was measured using the Coomassie Brilliant Blue method (Wang, Ji, et al. 2021). Total chlorophyll content was assessed using the direct extraction method (Homayoonzadeh et al. 2021). The kits used in the experiment were provided by Suzhou Grace Biotechnology Co. Ltd.

#### 2.3.3 *Amorphophallus* Yield and Glucomannan Content

During the maturation phase of *Amorphophallus*, all underground corms from each plot were harvested, and the surface soil was removed. The corms were weighed using an electronic balance to calculate the corm yield per unit area. The content of glucomannan in the corms was determined using the 3,5‐dinitrosalicylic acid method (Qi et al. 2023). After removing the bud and skin, corms were cut into 1‐mm thick slices and dried at 80°C for 48 h. After grinding, a powder with a particle diameter between 0.125 mm and 0.178 mm was reserved to measure KGM and starch content. Samples from three individual plants were mixed to form one biological replicate, and three biological replicates were used for KGM measurements. Approximately 0.1 g of konjac powder was added to 25 mL of 0.1 M formic acid sodium hydroxide buffer and left stirring at 30°C to dissolve and swell for 4 h. The volume was fixed to 50 mL and centrifuged at 4000 rpm for 20 min. Then, 5 mL of extract solution (supernatant) was added to 2.5 mL of 3 M sulfuric acid, sealed, and kept in a boiling water bath for 1.5 h. After cooling, 2.5 mL of 6 mol/L sodium hydroxide solution was added, and the volume was adjusted to 25 mL with distilled water; this solution was used for hydrolysis. Subsequently, 2 mL of extract was taken, hydrolysis solution and distilled water were added in a 25 mL volumetric flask, 1.5 mL of 1% 3,5‐dinitrosalicylic acid was added and kept in a water bath for 5 min; the volume was adjusted with distilled water, and the absorbance value at 550 nm was measured spectrophotometrically (UV‐2500, Shimadzu, Kyoto, Japan). Glucose was used for the standard curve, and distilled water served as a blank. Glucomannan content was calculated as follows:

$$\text{Konjac glucomannan content}\;\left(\%\right)=\frac{E\left(5T-T0\right)\times 50}{m\times \left(1-w\right)\times 1000}\times 100$$

*E* is the ratio of the molecular weight of glucose and mannose produced by hydrolysis of glucomannan, calculated as 0.9; *T* = the weight of glucose (mg) in the glucomannan hydrolysate as found on the standard curve; *T*0 = the weight of glucose (mg) in the glucomannan extract solution as found on the standard curve; *m* = the weight of the konjac powder sample (g); and *w* = the water content of the konjac powder sample (%).

### 2.4 Statistical Analyses

All experimental data were collected from 2022 to 2023 and were presented as the mean ± standard error of three replicates. Data were analyzed using ANOVA followed by Duncan's multiple range test (SPSS 21.0). Two significance levels, *p* < 0.05 and *p* < 0.01, were set for the analysis. Figures were generated using Origin 2017, and heat maps were created using TBtools v2.056.

## 3 Results

### 3.1 Analysis of Variance

**FIGURE 2:**
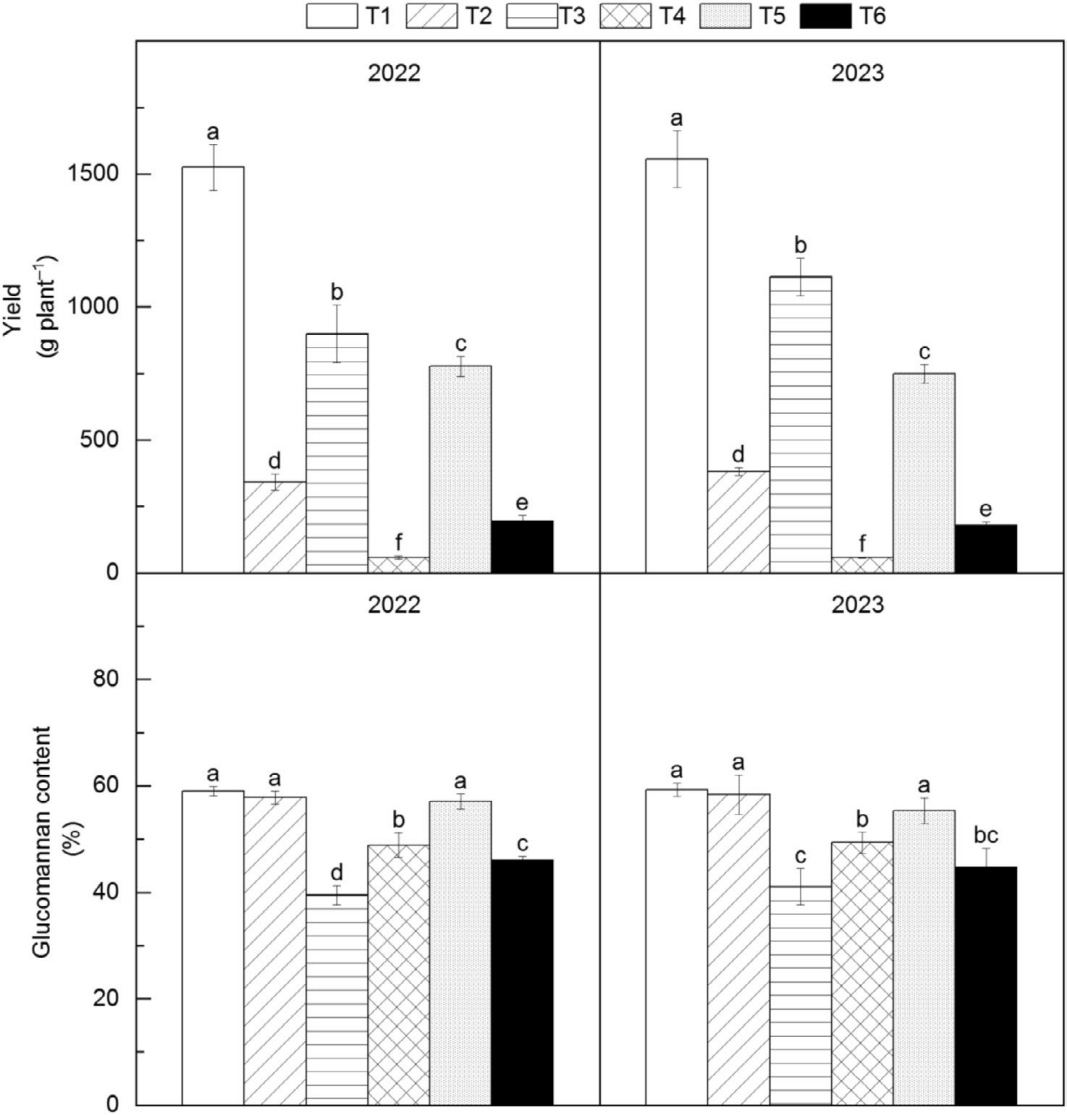
Changes in corm yield of different *Amorphophallus bulbifer* reproductive materials at the new corm formation stage, the bulking stage, and the mature stage in 2022 and 2023. T1: Two‐year‐old corms, T2: Bulbils, T3: One‐year‐old corms propagated from bulbils, T4: Seeds, T5: One‐year‐old corms propagated from seeds, T6: Tissue‐cultured seedlings. Different lowercase letters indicate statistical differences among treatments at *p* < 0.05.

**FIGURE 3:**
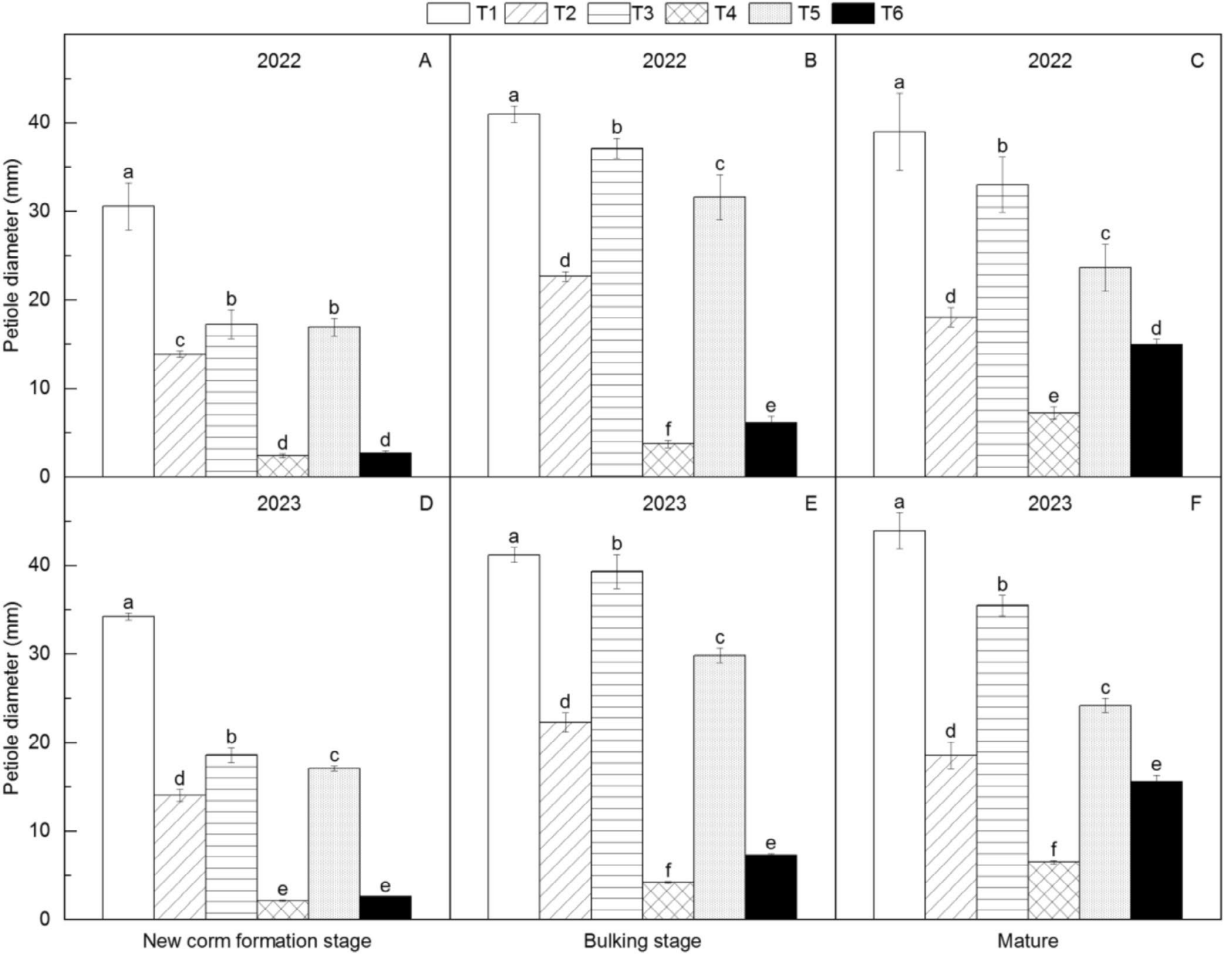
Changes in petiole base diameter of different *Amorphophallus bulbifer* reproductive materials at the new corm formation stage, the bulking stage, and the mature stage in 2022 and 2023. T1: Two‐year‐old corms, T2: Bulbils, T3: One‐year‐old corms propagated from bulbils, T4: Seeds, T5: One‐year‐old corms propagated from seeds, T6: Tissue‐cultured seedlings. Different lowercase letters indicate statistical differences among treatments at *p* < 0.05.

**FIGURE 4:**
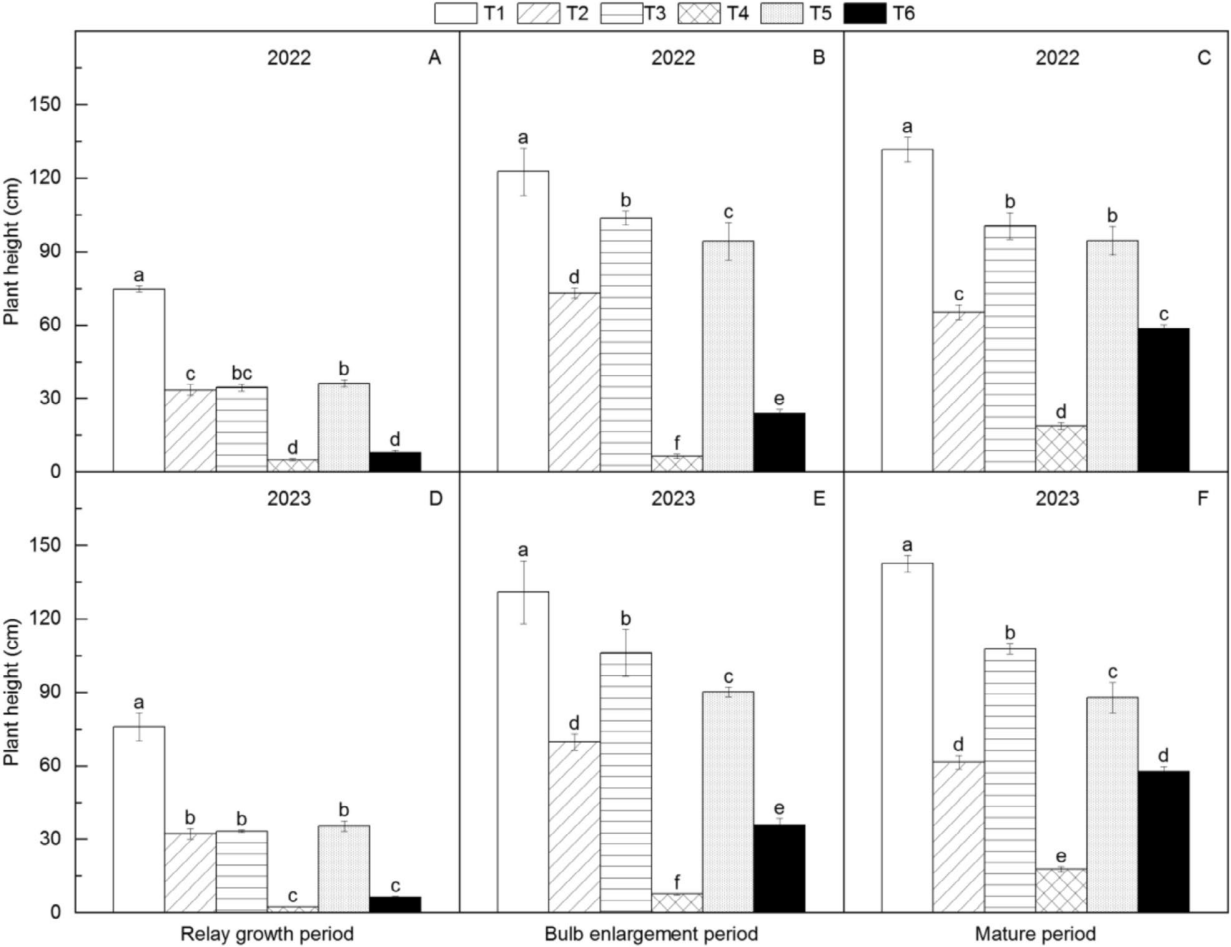
Changes in plant height of different *Amorphophallus bulbifer* reproductive materials at the new corm formation stage, the bulking stage, and the mature stage in 2022 and 2023. T1: Two‐year‐old corms, T2: Bulbils, T3: One‐year‐old corms propagated from bulbils, T4: Seeds, T5: One‐year‐old corms propagated from seeds, T6: Tissue‐cultured seedlings. Different lowercase letters indicate statistical differences among treatments at *p* < 0.05.

**FIGURE 5:**
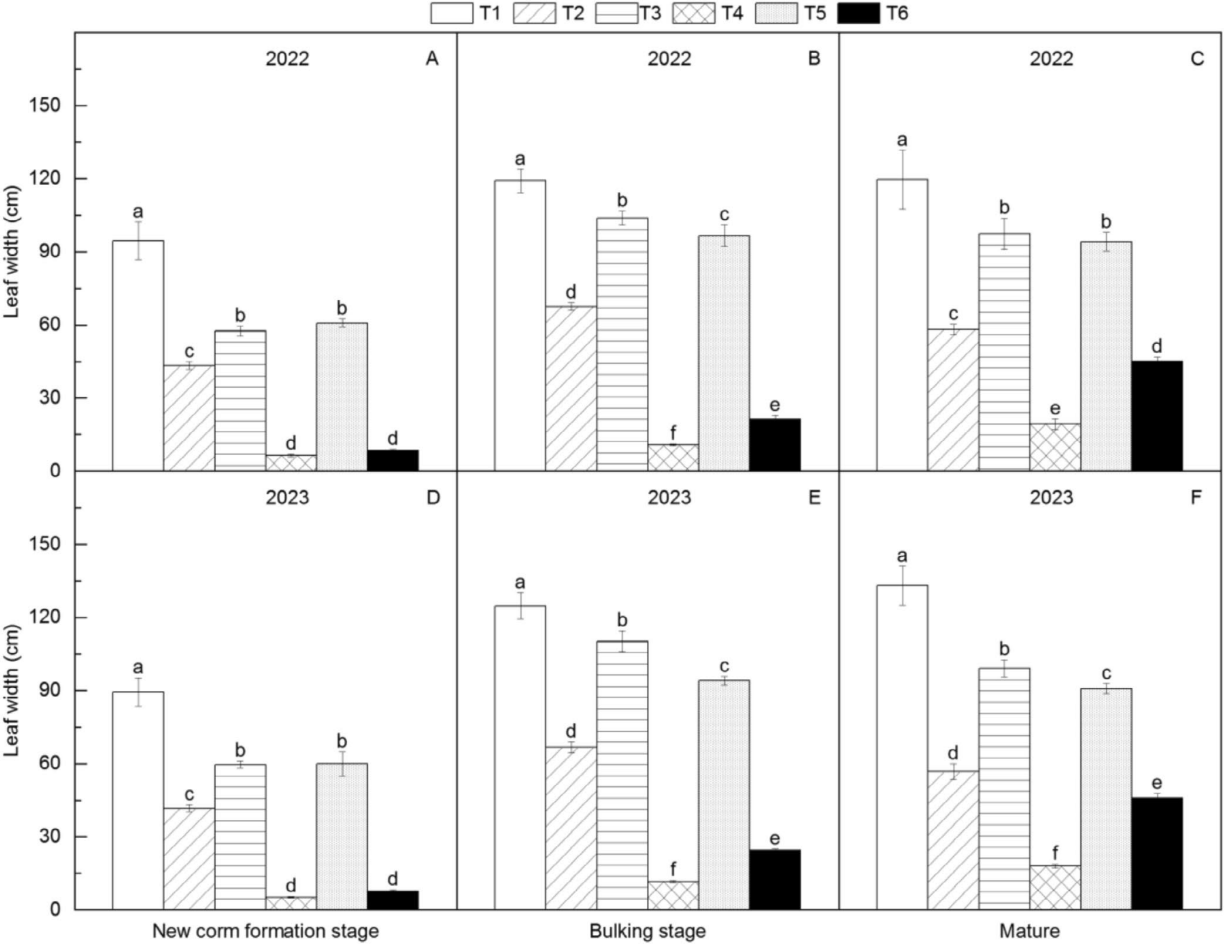
Changes in leaf width of different *Amorphophallus bulbifer* reproductive materials at the new corm formation stage, the bulking stage, and the mature stage in 2022 and 2023. T1: Two‐year‐old corms, T2: Bulbils, T3: One‐year‐old corms propagated from bulbils, T4: Seeds, T5: One‐year‐old corms propagated from seeds, T6: Tissue‐cultured seedlings. Different lowercase letters indicate statistical differences among treatments at *p* < 0.05.

**FIGURE 6:**
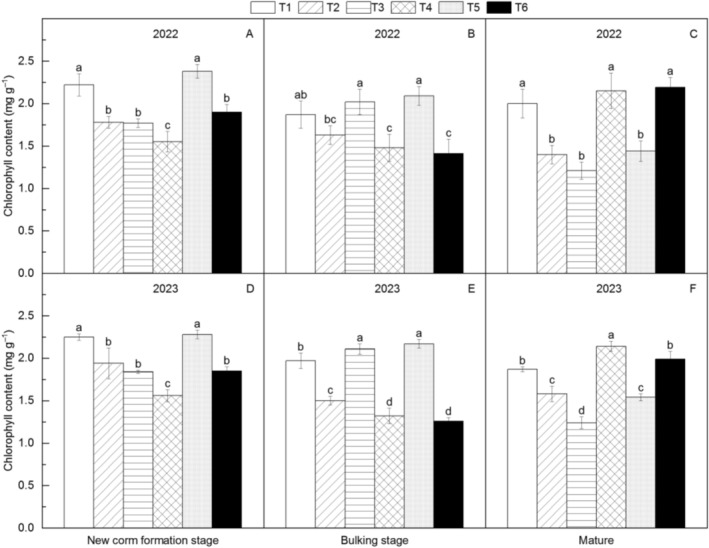
Changes in chlorophyll content of different *Amorphophallus bulbifer* reproductive materials at the new corm formation stage, the bulking stage, and the mature stage in 2022 and 2023. T1: Two‐year‐old corms, T2: Bulbils, T3: One‐year‐old corms propagated from bulbils, T4: Seeds, T5: One‐year‐old corms propagated from seeds, T6: Tissue‐cultured seedlings. Different lowercase letters indicate statistical differences among treatments at *p* < 0.05.

**FIGURE 7:**
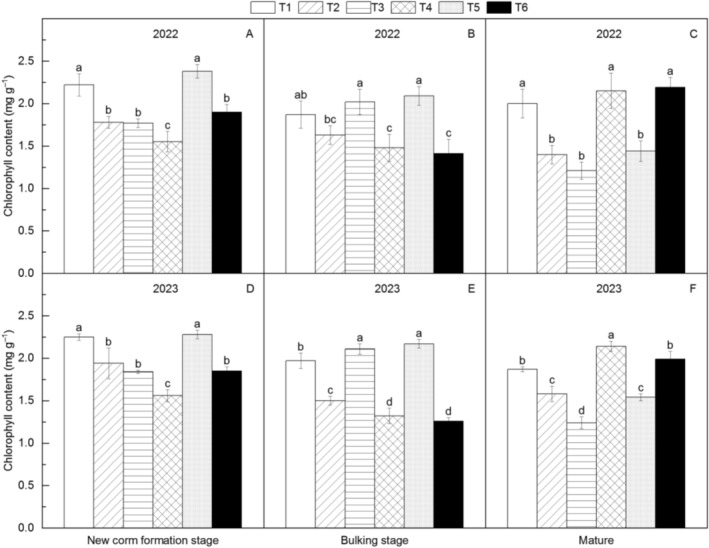
Changes in net photosynthetic rate of different *Amorphophallus bulbifer* reproductive materials at the new corm formation stage, the bulking stage, and the mature stage in 2022 and 2023. T1: Two‐year‐old corms, T2: Bulbils, T3: One‐year‐old corms propagated from bulbils, T4: Seeds, T5: One‐year‐old corms propagated from seeds, T6: Tissue‐cultured seedlings. Different lowercase letters indicate statistical differences among treatments at *p* < 0.05.

**FIGURE 8:**
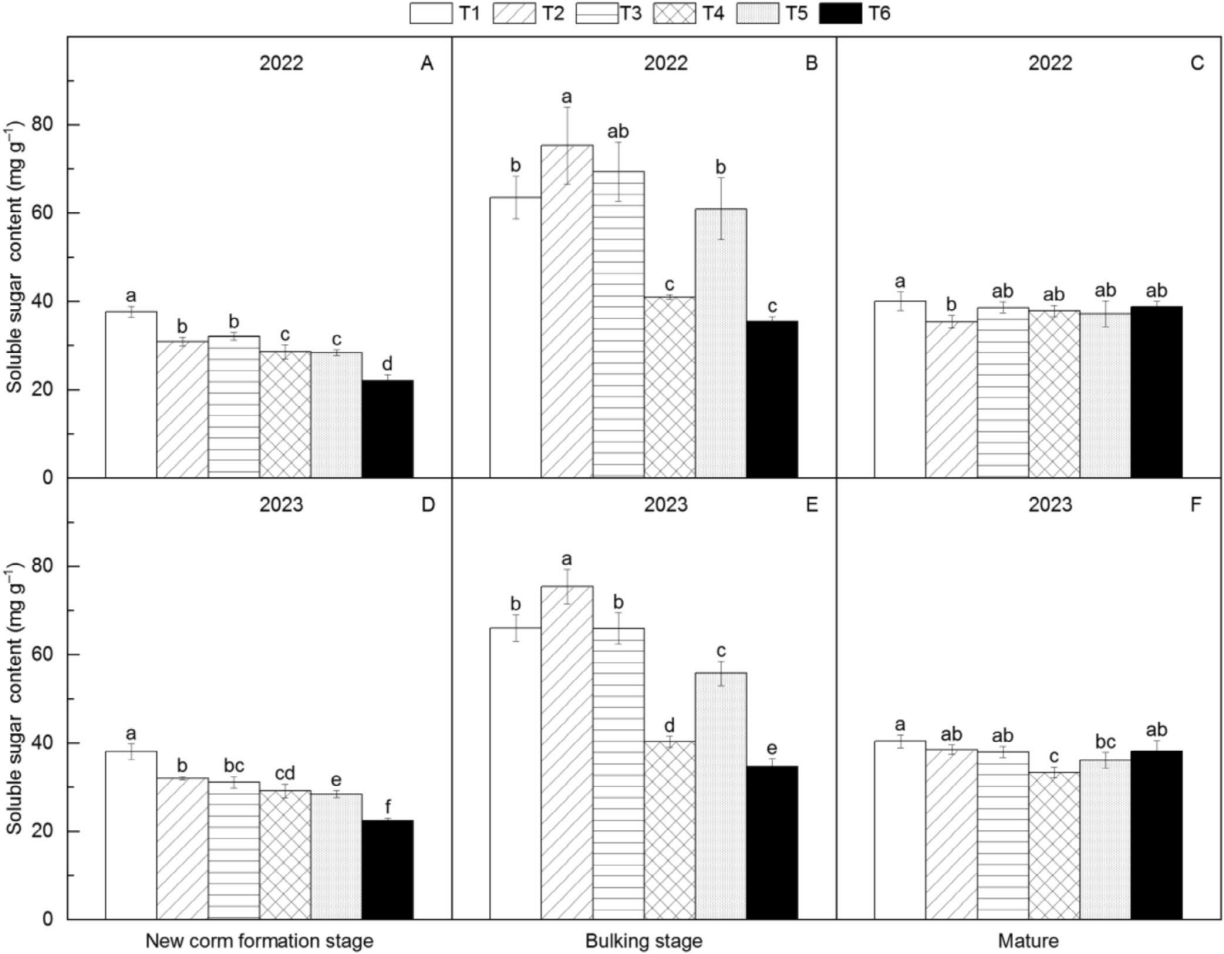
Changes in soluble sugar content of different *Amorphophallus bulbifer* reproductive materials at the new corm formation stage, the bulking stage, and the mature stage in 2022 and 2023. T1: Two‐year‐old corms, T2: Bulbils, T3: One‐year‐old corms propagated from bulbils, T4: Seeds, T5: One‐year‐old corms propagated from seeds, T6: Tissue‐cultured seedlings. Different lowercase letters indicate statistical differences among treatments at *p* < 0.05.

**FIGURE 9:**
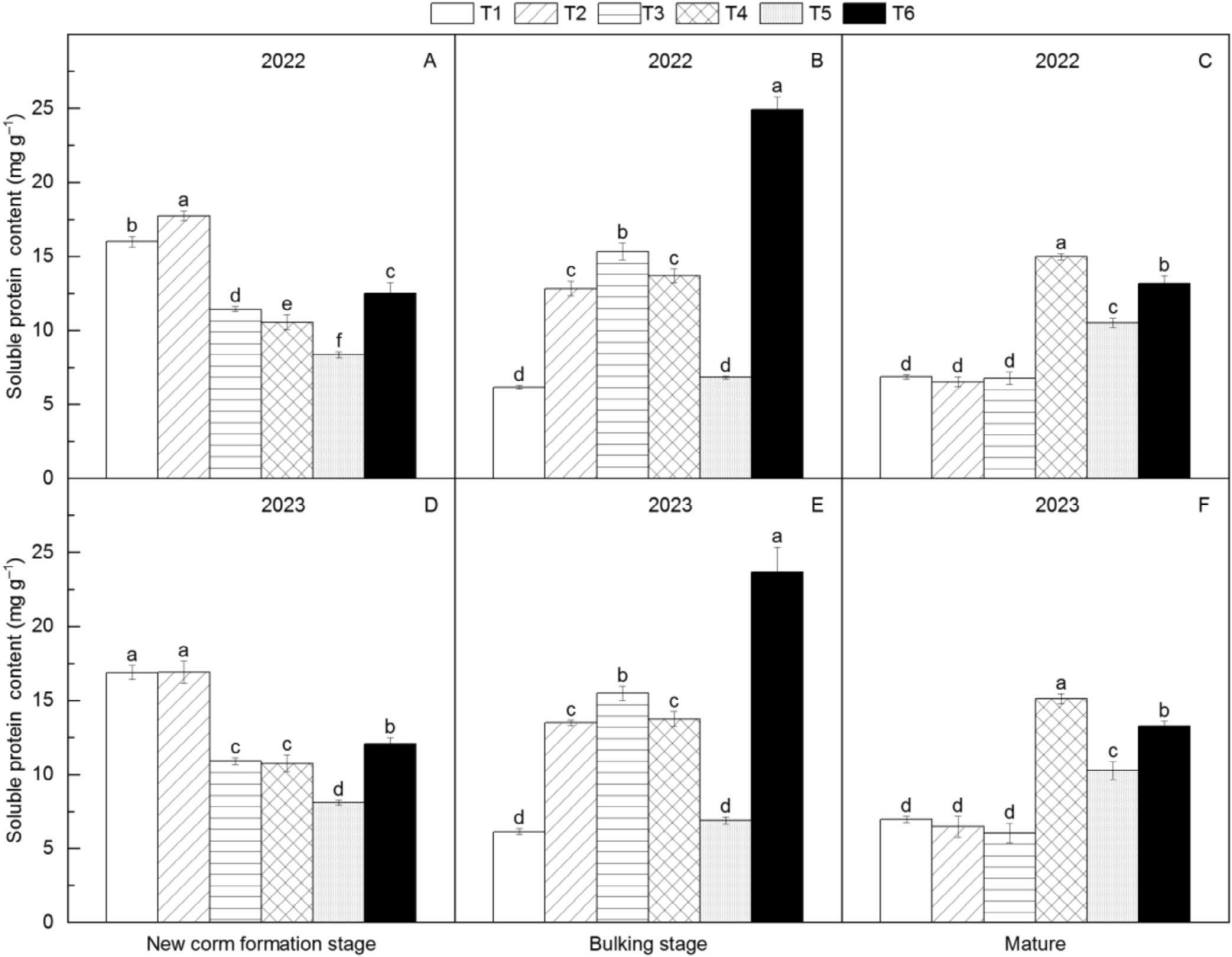
Changes in soluble protein content of different *Amorphophallus bulbifer* reproductive materials at the new corm formation stage, the bulking stage, and the mature stage in 2022 and 2023. T1: Two‐year‐old corms, T2: Bulbils, T3: One‐year‐old corms propagated from bulbils, T4: Seeds, T5: One‐year‐old corms propagated from seeds, T6: Tissue‐cultured seedlings. Different lowercase letters indicate statistical differences among treatments at *p* < 0.05.

The treatment of reproductive materials had a significant impact on various indicators of *Amorphophallus* (Table 1). The interaction between year and reproductive material treatment significantly influenced yield, petiole diameter, and soluble protein content during the new corm formation stage, as well as plant height and soluble protein content at maturity. Further analysis revealed that the patterns of differences in various indicators of *Amorphophallus* among the different reproductive material treatments were generally consistent over the 2 years (Figures 2, 3, 4, 5, 6, 7, 8, 9).

**TABLE 1:** The ANOVA for the effect of year (Y) and reproductive materials (R) on the indicator of *Amorphophallus muelleri*.

Treatments	Total yield	Glucomannan	Petiole base diameter			Plant height			Leaf width			Chlorophyll			Soluble sugar			Soluble protein			Net photosynthetic rate		
	t ha^−1^	%	S1	S2	S3	S1	S2	S3	S1	S2	S3	S1	S2	S3	S1	S2	S3	S1	S2	S3	S1	S2	S3
Year (Y)	ns	ns	ns	ns	ns	ns	ns	ns	ns	ns	ns	ns	ns	ns	ns	ns	ns	ns	ns	ns	ns	ns	ns
Reproductive materials (R)	**	**	**	**	**	**	**	**	**	**	**	**	**	**	**	**	**	**	**	**	**	**	**
Y*R	*	ns	*	ns	ns	ns	ns	**	ns	ns	ns	ns	ns	ns	ns	ns	*	*	ns	ns	ns	ns	ns

*Note:* Asterisks indicate a significant difference at different treatments (*p < 0.05, **p < 0.01). S1, S2, and S3 mean relay growth period, bulb enlargement period, and mature period, respectively.

### 3.2 *Amorphophallus* Corm Yield and Glucomannan Content

Significant differences in corm yield were observed among propagation materials (Figure 2). T1 yielded the highest (1539.19 g plant^−1^), followed by T3 (1005.31 g plant^−1^), T5 (762.82 g plant^−1^), T2 (361.74 g plant^−1^), T6 (186.75 g plant^−1^) and T4 (58.16 g plant^−1^). Glucomannan content was highest in T1 (59.17%), T2 (58.12%), and T5 (56.16%), significantly exceeding other treatments (T4:49.12%, T6:45.37%, T3:40.29%).

### 3.3 Dynamic Changes in Morphological Characteristics of *Amorphophallus*

Across all growth stages, T1 consistently showed the largest petiole diameter, plant height, and leaf area, while T4 was smallest (Figures 3, 4, 5). Morphological parameters increased significantly from new corm formation to bulking stage but stabilized thereafter. Among intermediate‐weight materials (~20 g), T3 > T5 > T2 > T6 for size. Differences between T3/T5/T2 widened with growth, while T2/T6 differences narrowed.

### 3.4 Chlorophyll Content in *Amorphophallus* Leaves

Significant variations in chlorophyll content were observed across treatments and growth stages (Figure 6). During the new corm formation stage, chlorophyll content was highest in treatment T5, exceeding T4 by 49.36%. In the bulking stage, T5 maintained the highest chlorophyll levels, surpassing T6 by 58.96%, with the overall ranking being T5 > T3 > T1 > T2 > T4 > T6. At maturity, treatments T4, T1, and T6 exhibited the highest chlorophyll content, with T4 showing 74.80% greater content than T3.

### 3.5 Net Photosynthetic Rate in *Amorphophallus* Leaves

The Pn demonstrated stage‐dependent patterns (Figure 7). Minimal differences were observed during the new corm formation stage, with T3 showing only an 8.26% advantage over T6. In the bulking stage, T6 displayed the highest Pn (24.74% greater than T2), with the treatment ranking being T6 > T4 > T3 > T5 > T1 > T2. This pattern persisted at maturity, where T6 maintained the highest Pn (97.18% above T2), followed by the order T6 > T4 > T5 > T1 > T3 > T2.

### 3.6 Soluble Sugar Content in *Amorphophallus* Leaves

Soluble sugar content exhibited distinct treatment hierarchies across growth phases (Figure 8). At the new corm formation stage, T1 contained the highest soluble sugar levels, exceeding T6 by 70.19%, with the full ranking being T1 > T3 > T2 > T4 > T5 > T6. During the bulking stage, T2 showed the highest concentration (114.91% greater than T6), followed by the sequence T2 > T3 > T1 > T5 > T4 > T6. At maturity, T1 maintained the highest soluble sugar content (12.98% above T4), with no significant differences among other treatments.

### 3.7 Soluble Protein Content in *Amorphophallus* Leaves

Soluble protein content varied substantially among treatments (Figure 9). In the new corm formation stage, T2 contained the highest protein levels (110.45% greater than T5), with the treatment order being T2 > T1 > T6 > T3 > T4 > T5. During the bulking stage, T6 showed the highest protein content (253.94% above T1), ranked as T6 > T3 > T4 > T2 > T5 > T1. At maturity, T4 exhibited the maximum protein levels (134.71% higher than T3), with significant differences among the top treatments (T4 > T6 > T5).

### 3.8 Correlation Analysis

**FIGURE 10:**
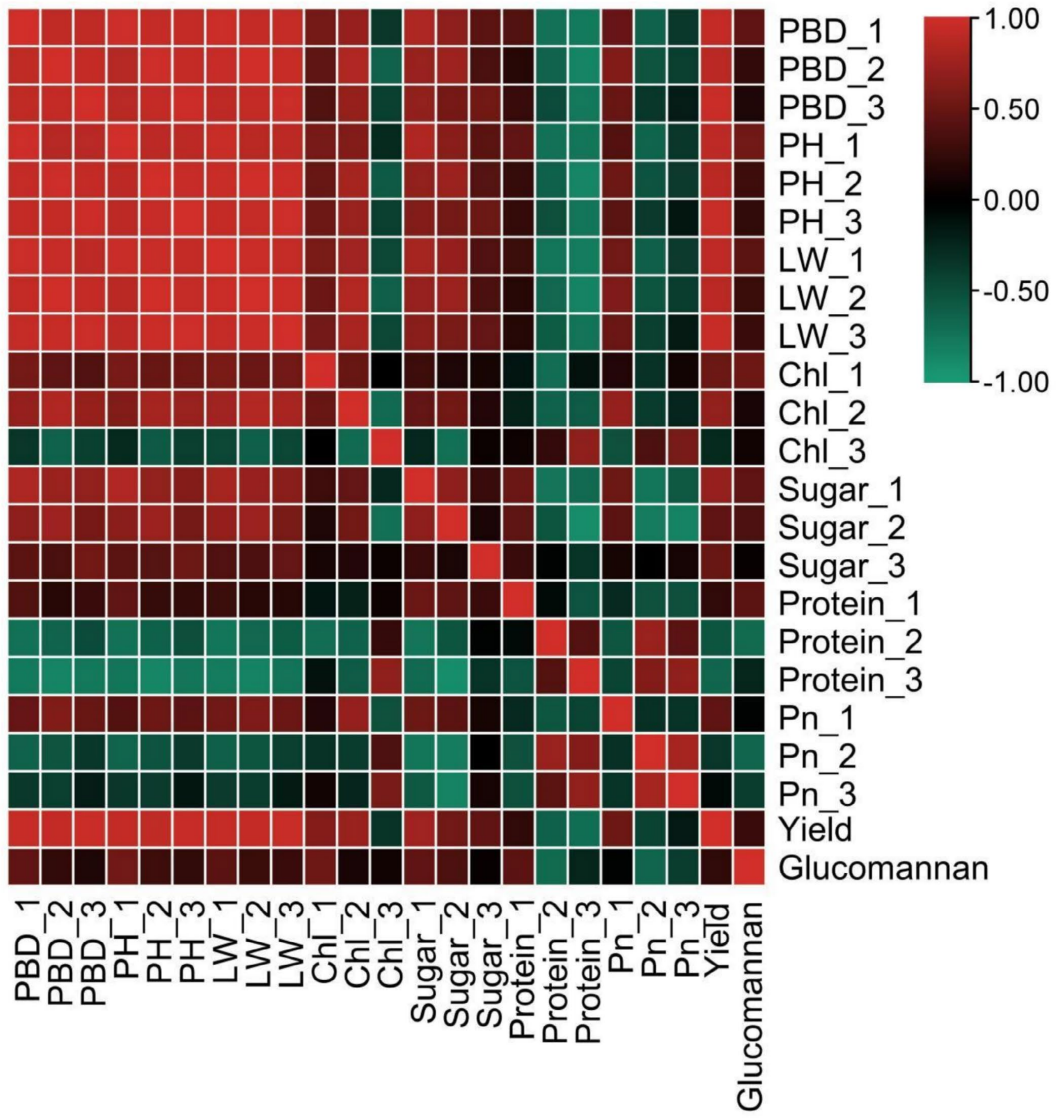
Heat map of correlation analysis among various indicators. Red represents positive correlation and green represents negative correlation. Petiole base diameter (PBD); Plant height (PH); Leaf width (LW); Chlorophyll content (Chl); Soluble sugar content (Sugar); Soluble protein content (Protein); Net photosynthetic rate (Pn). The numbers 1, 2, and 3 after the underline represent the Relay growth period, bulb enlargement period and mature period, respectively.

Overall, the yield exhibited a highly significant positive correlation with petiole diameter, plant height, leaf area, and soluble sugar content across the three growth stages, as well as with chlorophyll content in the new corm formation and bulking stages, and net photosynthetic rate during the new corm formation stage (Figure 10). Conversely, a highly significant negative correlation was observed with the soluble protein content in the new corm formation and bulking stages, as well as with the net photosynthetic rate in the bulking stage. The glucomannan content exhibited a highly significant positive correlation with petiole diameter, plant height, leaf area, chlorophyll content, and both soluble protein and soluble sugar content in the new corm formation stage, as well as with soluble sugar content during the bulking stage. Conversely, it showed a highly significant negative correlation with soluble protein content in the new corm formation stage and with net photosynthetic rate in both the new corm formation and maturity stages.

## 4 Discussion

### 4.1 Differences in Aboveground Morphological Variations Among Different Reproductive Materials

The aboveground and underground parts of plants frequently engage in energy and material exchange, and the morphology of the aboveground portions largely determines the corms' yield (Szechynska‐Hebda et al. 2022). Key morphological indicators such as plant height, petiole diameter, and leaf area reflect the characteristics of the aboveground morphology, making them essential criteria for the screening of reproductive materials (Enkhbat et al. 2022). Additionally, leaf area plays a critical role in determining the ability of *Amorphophallus* leaves to capture light energy, which is crucial for photosynthesis and overall plant productivity (Qin et al. 2019). In this study, from the new corm formation stage to the bulking stage, the aboveground parts of *A. muelleri* exhibited rapid growth, followed by a stabilization phase, which is consistent with previous research findings (Huifang et al. 2024). After undergoing a “relay growth” process, the leaf area and petiole length increased significantly, leading to a rapid expansion of the new tubers. Approximately 1 month later, the aboveground growth reached its peak, resulting in a substantial accumulation and translocation of photosynthetic products to the new tubers.

This study found that the greater the weight of the reproductive materials, the larger the aboveground morphological structures. For example, the aboveground vegetative parts were largest under the treatment of 2‐year‐old corms (approximately 150 g), while the smallest were observed under seeds treatment (approximately 0.1 g). This may be related to the fact that heavier reproductive materials provide more energy for the growth and development of new organs (Putri et al. 2023). When comparing materials of equal weight (approximately 20 g), the size order of the aboveground morphological structures was as follows: 1‐year‐old corms propagated from bulbils > 1‐year‐old corms propagated from seeds > bulbils. This demonstrates that the growth performance of corms as reproductive materials is superior to that of bulbils. Additionally, this study found that the aboveground growth was slow during the early stages under tissue‐cultured seedlings treatment. The reasons for this phenomenon require further investigation.

### 4.2 Differences in Physiological Indicators Among Different Reproductive Materials

Chlorophyll, as the pigment responsible for capturing and absorbing light energy in plants, serves as a crucial indicator of photosynthetic capacity. The net photosynthetic rate is influenced by both genetic factors and environmental conditions, and it is closely related to the plant's photosynthetic ability and yield. Consequently, it is often used as a vital criterion for evaluating plant adaptability. This study found that the changes in chlorophyll content from the new corm formation stage to the bulking stage were generally consistent with the growth rate of the aboveground parts under all treatments, which aligns with findings reported in other crops (Yang et al. 2023; Zheng et al. 2022). Interestingly, from the bulking stage to maturity, the chlorophyll content in 1‐year‐old corms propagated from bulbils or seeds exhibited a significant decline, whereas those propagated from seeds and tissue‐cultured seedlings showed a notable increase in chlorophyll content. This suggests that during this period, the aboveground parts of 1‐year‐old corms from bulbil or seed propagation began to show signs of aging, leading to chlorophyll degradation. In contrast, the aboveground parts of seeds and tissue‐cultured seedlings did not exhibit senescence, as chlorophyll synthesis and accumulation continued. This phenomenon is likely associated with the relatively slow growth and prolonged time required to reach peak growth observed in the seeds and tissue‐cultured seedlings treatments, leading to a delayed peak in chlorophyll synthesis and accumulation compared to the other treatments (Shi et al. 2023; Wang, Kang, et al. 2021). Consequently, this resulted in the highest chlorophyll content observed during maturation for seeds and tissue‐cultured seedlings treatments. Additionally, during maturity, the 2‐year‐old corms treatment maintained higher chlorophyll levels, possibly due to its capacity to store more nutrients, which facilitated the early development of a stronger nutrient base. This provided the plant with additional energy and resources for chlorophyll synthesis (de Bang et al. 2021), though further studies are necessary to elucidate the underlying mechanisms.

In this study, the net photosynthetic rates among different treatments were relatively similar during the new corm formation stage. The treatment with 1‐year‐old corms propagated from bulbils had the highest net photosynthetic rate, which was only 8.26% higher than that of the lowest rate observed in the tissue‐cultured seedlings. This phenomenon may be attributed to the experimental conditions, under which all propagation materials were able to reach their net photosynthetic rate ceiling (approximately 20 μmol m^−2^ s^−1^). Therefore, identifying methods to further enhance the net photosynthetic rate of *A. bulbifer* to increase corm yield is a key area for future research. It is noteworthy that from the bulking stage to maturity, the net photosynthetic rates of all treatments significantly decreased due to plant senescence (Liu et al. 2022). However, the tissue‐cultured seedlings maintained relatively high net photosynthetic rates, which may be attributed to their slow growth and delayed chlorophyll degradation.

Soluble sugars are a vital energy source for plant growth and development, reflecting the metabolic efficiency of the plant (Choudhary et al. 2022). In this study, as *A. bulbifer* grew, the soluble sugar content in all treatments followed a trend of initially increasing and then decreasing, except for the tissue‐cultured seedlings, for which the soluble sugar content continuously increased. This indicates that the metabolic efficiency of *A. bulbifer* initially increases and then decreases as the plant matures, which is consistent with previous reports (Huifang et al. 2024). In the new corm formation stage, the highest soluble sugar content was found in the 2‐year‐old corms treatment. From the new corm formation stage to the bulking stage, the greatest increases in soluble sugar content were noted in the 1‐year‐old corms propagated from bulbils or seeds and the bulbils treatments. From the bulking stage to maturity, the soluble sugar content decreased by 12.45% in the seeds treatment, whereas it increased by 9.77% in the tissue‐cultured seedlings treatment. These findings suggest that the metabolic efficiency varies among treatments, with the 2‐year‐old corms exhibiting the highest rate, followed by the 1‐year‐old corms propagated from bulbils or seeds and the bulbils. Conversely, the seed and tissue‐cultured seedlings treatments displayed the lowest metabolic efficiency. This trend is consistent with the growth patterns observed in the aboveground parts of *A. bulbifer* .

Soluble proteins are important nutrients and osmotic regulators in plants (Alghamdi et al. 2022). The soluble proteins in leaves primarily originate from photosynthetic enzymes, and they also serve as indicators of the photosynthetic capacity of the leaves (Amer et al. 2021). In this study, in the new corm formation stage, the highest soluble protein content was observed in the bulbils and 2‐year‐old corms treatments. In the bulking stage, both treatments experienced the greatest reduction in soluble protein content. At maturity, the soluble protein content in the 2‐year‐old corm treatment increased by 12.45%, whereas in the bulbil treatment, the soluble protein content significantly decreased by 50.68%. Another noteworthy treatment is the tissue‐cultured seedlings, which exhibited a 97.52% increase in soluble protein content from the new corm formation stage to the bulking stage, followed by a decrease of 45.50% by maturity. The fluctuations in soluble protein content are related to the genetic characteristics of the propagation materials and their adaptability to the environment (Iqbal et al. 2020). However, the mechanisms behind these variations among the treatments require further investigation.

### 4.3 Yield and Glucomannan Content

The yield and glucomannan content of *A. bulbifer* corms is an important economic trait for assessing the feasibility of various propagation materials (Nurshanti et al. 2023). In this study, the highest yield was observed in the 2‐year‐old corms treatment, which is consistent with our hypothesis. The 2‐year‐old corms weighed 150 g, providing a greater supply of nutrients for the growth of the aboveground parts and new corms. Our morphological and physiological analyses also indicated that the growth rate of the 2‐year‐old corms was the fastest, and their nutrient content remained significantly higher than that of other treatments. Furthermore, they were able to maintain elevated levels of chlorophyll and soluble sugars in the later growth stages. However, the substantial weight of the 2‐year‐old corms poses challenges for storage, transportation, and planting, and their bulking ratio is approximately 10.26 times lower than that of all other treatments. The yield from 1‐year‐old corms propagated from bulbils treatment ranks second, with a weight of only 20 g and a bulking ratio of 50.27 times, making it more suitable for practical applications compared to the 2‐year‐old corms. Notably, when bulbils are used as propagation material, their bulking ratio is 17.10 times, demonstrating the feasibility of using bulbils as the initial propagation material for producing 1‐year‐old corms, which can serve as marketable *A. bulbifer* corms. Although tissue‐cultured seedlings can provide a large number of genetically stable and uniformly growing seedlings in a short period (Nurshanti et al. 2023), the corms yield in seedlings tissue‐cultured seedlings treatment is only 9.34 kg ha^−1^. Therefore, further research is needed to improve the tissue‐cultured seedlings technology. The glucomannan content in 1‐year‐old corms propagated from seeds treatment was found to be higher than that of 1‐year‐old corms propagated from bulbils. Consequently, the total amount of glucomannan in *A. bulbifer* corms obtained from 1‐year‐old corms propagated from seeds (428.40 g plant^−1^) exceeded that of those from 1‐year‐old corms propagated from bulbils (405.04 g plant^−1^). Therefore, from the perspective of glucan acquisition, annual tubers propagated by seed represent the optimal reproductive material. The lower glucomannan in T3 (1‐year corms from bulbils) despite higher yield suggests a trade‐off between biomass accumulation and specialized metabolite synthesis, possibly influenced by source‐sink relationships or developmental programming established early in the propagation material (Shenglin et al. 2020). However, the mechanisms underlying the differences in glucan content among different propagation methods require further investigation. The correlation analysis indicates that promoting the growth rate and size of the aboveground parts can enhance the corms yield and glucomannan content of *A. bulbifer* .

## 5 Conclusion

Two‐year‐old corms used as propagation material produced significantly higher yields than other propagation materials. However, they have the drawbacks of greater weight and a lower expansion coefficient. One‐year‐old corms propagated from bulbils or seeds also yielded reasonably high production levels. Considering the glucomannan content associated with each treatment, this study concludes that 1‐year‐old corms propagated from seeds are the optimal propagation material. However, the mechanisms underlying the differences in physiological indicators and glucomannan content among various propagation materials require further investigation.

## Author Contributions

X.S.: Investigation, formal analysis. L.J.: Investigation. Y.H.: Formal analysis, data curation. M.S.: Investigation, data curation. L.Z.: Writing – review and editing, writing – original draft, visualization, supervision, conceptualization. Y.L.: Data curation. G.Y.: Investigation. Y.S.: Methodology, investigation. X.W.: Validation, supervision, resources, methodology. Y.X.: Writing – review and editing, supervision, investigation.

## Conflicts of Interest

The authors declare no conflicts of interest.

## Data Availability

Data will be made available on request.
